# Post-graduate nephrology education in China: structure, workforce gaps, regional disparities, and the emerging role of critical care nephrology

**DOI:** 10.1080/0886022X.2026.2731711

**Published:** 2026-09-22

**Authors:** Yanni Wang, Jian Sun, Jianbo Qing, Suibi Yang, Xiaocong Jiang, Min Yang, Zhongheng Zhang

**Affiliations:** ^a^ The Second Department of Critical Care Medicine, the Second Affiliated Hospital of Anhui Medical University, Hefei, China; ^b^ Department of Emergency Medicine, Sir Run Run Shaw Hospital, Zhejiang University School of Medicine, Hangzhou, China; ^c^ Department of Critical Care Medicine, Lishui Central Hospital, Li Shui, China; ^d^ Department of Nephrology, Sir Run Run Shaw Hospital, Zhejiang University School of Medicine, Hangzhou, China; ^e^ Laboratory of Cardiopulmonary Resuscitation and Critical Illness, the Second Affiliated Hospital of Anhui Medical University, Hefei, China; ^f^ School of Medicine, Shaoxing University, Shaoxing, P.R. China; ^g^ Provincial Key Laboratory of Precise Diagnosis and Treatment of Abdominal Infection, Sir Run Run Shaw Hospital, Zhejiang University School of Medicine, Hangzhou, China; ^h^ Longquan Industrial Innovation Research Institute, Lishui, China

**Keywords:** Nephrology education, training pathways, workforce shortage, standardized residency training, critical care nephrology

## Abstract

Medical education systems vary widely across countries, yet the structure of medical and postgraduate training in China remains relatively unfamiliar to international readers. China has developed a relatively comprehensive pathway spanning undergraduate education, postgraduate programs, standardized residency training, and standardized specialty training, thereby shaping an evolving framework that aims to integrate clinical practice with scientific research. Within this framework, postgraduate nephrology education highlights both clinical training and research development. In recent years, Critical Care Nephrology has demonstrated notable strengths in the management of critical illness, blood purification, and multidisciplinary collaboration. This review provides an overview of the development, current status, and future trends in medical and postgraduate nephrology education in China, with a particular focus on the emerging role of Critical Care Nephrology.

## Graphical abstract

**Figure:**
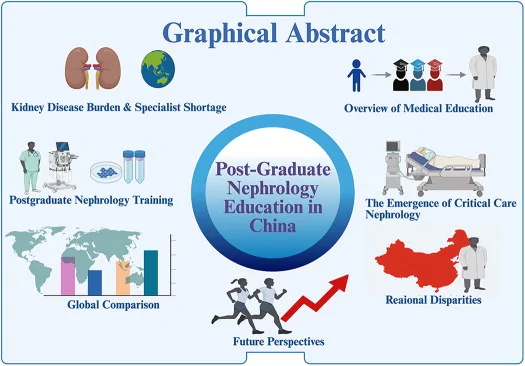

## 1. Introduction

### 1.1. Global burden of kidney disease and the shortage of nephrology specialists

Chronic kidney disease (CKD) has emerged as a global public health challenge, with its disease spectrum in China increasingly aligning with that of developed countries, which carries significant implications for healthcare systems [1]. It is estimated that approximately 850 million people worldwide are affected by CKD to varying degrees, contributing to its high prevalence, disability rates, and substantial treatment costs associated with progression to end-stage kidney disease (ESKD), thus placing a heavy burden on healthcare systems in many countries [2]. A joint statement from the International Society of Nephrology (ISN) and other organizations highlights that kidney disease is one of the leading non-communicable diseases, causing premature death, and calls for its inclusion in the World Health Organization’s key prevention and control initiatives.

Despite the growing burden of kidney disease, the shortage of nephrology specialists is becoming increasingly severe worldwide, particularly in resource-limited regions like Africa, where inadequate healthcare infrastructure exacerbates diagnostic and treatment challenges [3,4]. Many low-income countries lack structured nephrology training programs, leading to a significant shortfall in the development of local talent, which further intensifies the unequal distribution of healthcare resources. In regions such as North America and East Asia, although the density of nephrologists is higher, significant disparities remain between countries, and access to services like home hemodialysis (HHD) continues to be limited [5]. The American Society of Nephrology (ASN) has made efforts to alleviate the workforce crisis by establishing specialized training programs, such as transplant nephrology fellowships, but recruitment difficulties persist as a widespread issue in the field [6,7]. Therefore, the training and equitable distribution of nephrology specialists globally has become a critical factor in enhancing kidney disease diagnosis and treatment capabilities, as well as in achieving sustainable development goals.

### 1.2. Historical development of nephrology education in China

**Figure 1.**
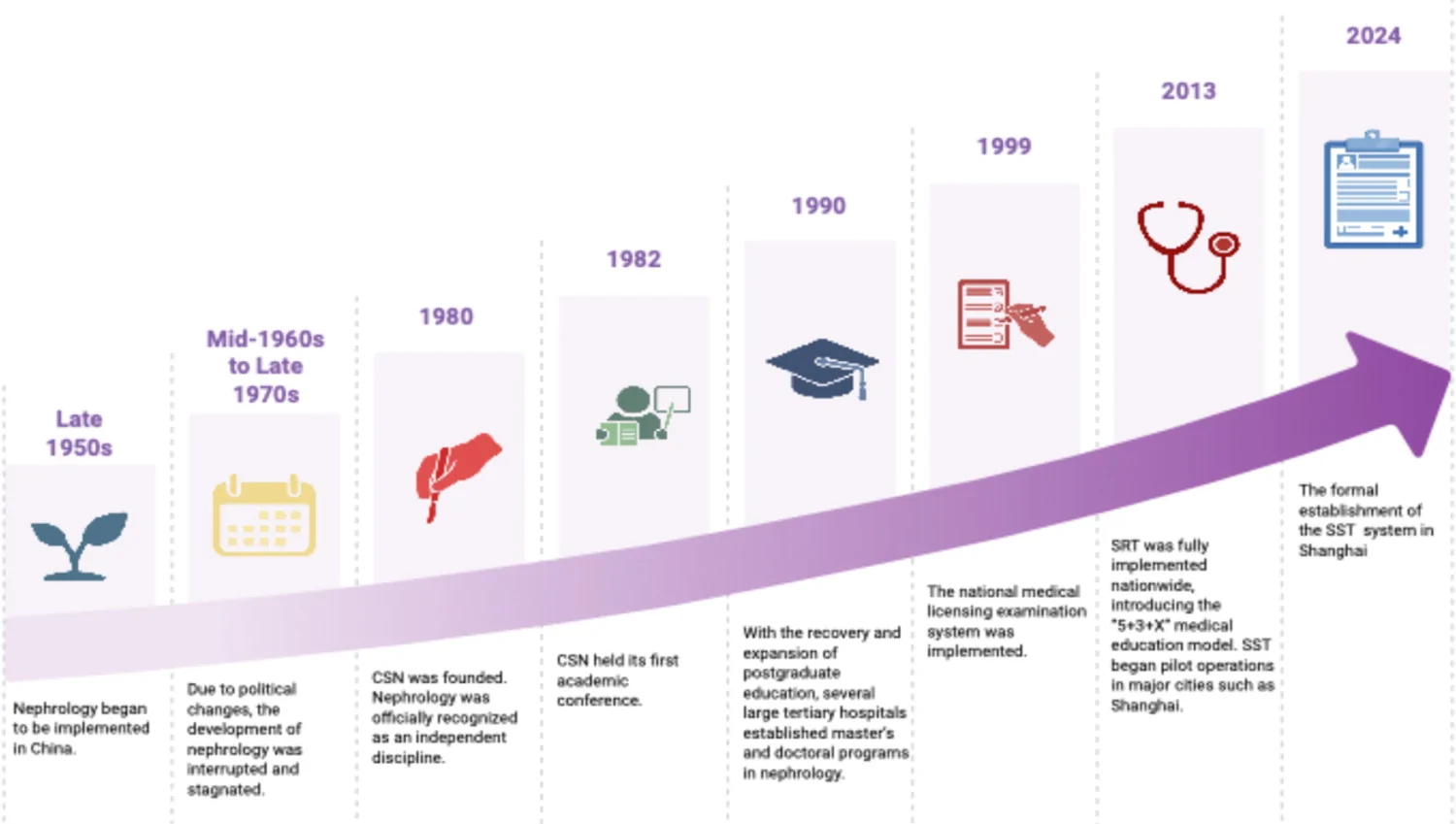
Timeline of Nephrology Education in China.

Nephrology in China began in the late 1950s, but it was not until the mid-1980s that it became an independent specialty, establishing connections with the international nephrology community. Due to the country’s large population and vast territory, nephrologists in China face significant responsibilities and considerable challenges. Early nephrologists in China were primarily internal medicine physicians who transitioned into nephrology through a mentorship system, lacking a structured specialty training framework. In medical education, China has established a relatively comprehensive national framework encompassing undergraduate education, postgraduate programs, standardized residency training (SRT), and standardized specialty training (SST). Figure 1 highlights key milestones in the development of nephrology education, including the founding of the CSN, the implementation of the medical licensing system, and the introduction of SRT and SST. However, heterogeneity remains in curriculum delivery, assessment practices, and training quality across institutions and regions [8]. With the expansion of postgraduate education, improving the overall quality of postgraduate teaching has become a core task in the reform and development of medical education in China [9,10]. Based on an analysis of literature, nephrology clinical research papers increased by an average of 11.55 per year, while basic research papers grew by 4.55 per year from 2001 to 2011, highlighting the substantial rise in clinical research output [11]. In recent years, the application of precision nephrology and digital intelligent technologies has become a major focus, offering new directions for personalized diagnosis and treatment, as well as innovations in education [12]. Critical Care Nephrology (CCN), as an emerging sub-specialty, has increasingly focused on blood purification and multidisciplinary collaboration, reflecting the dynamic development of nephrology education in China [13,14].

### 1.3. Purpose and significance of this review

This review aims to provide a comprehensive analysis of the current status and development of postgraduate nephrology education in China, with particular emphasis on the emerging role of CCN as a subspecialty, alongside the challenges it faces in clinical training and research capacity building. The medical education system in China differs significantly from those in other countries, and there is a lack of systematic analysis on postgraduate nephrology education in China. By comparing China’s system with international counterparts, this review provides valuable insights for the internationalization of future educational models.

## 2. Overview of medical education in China

**Figure 2.**
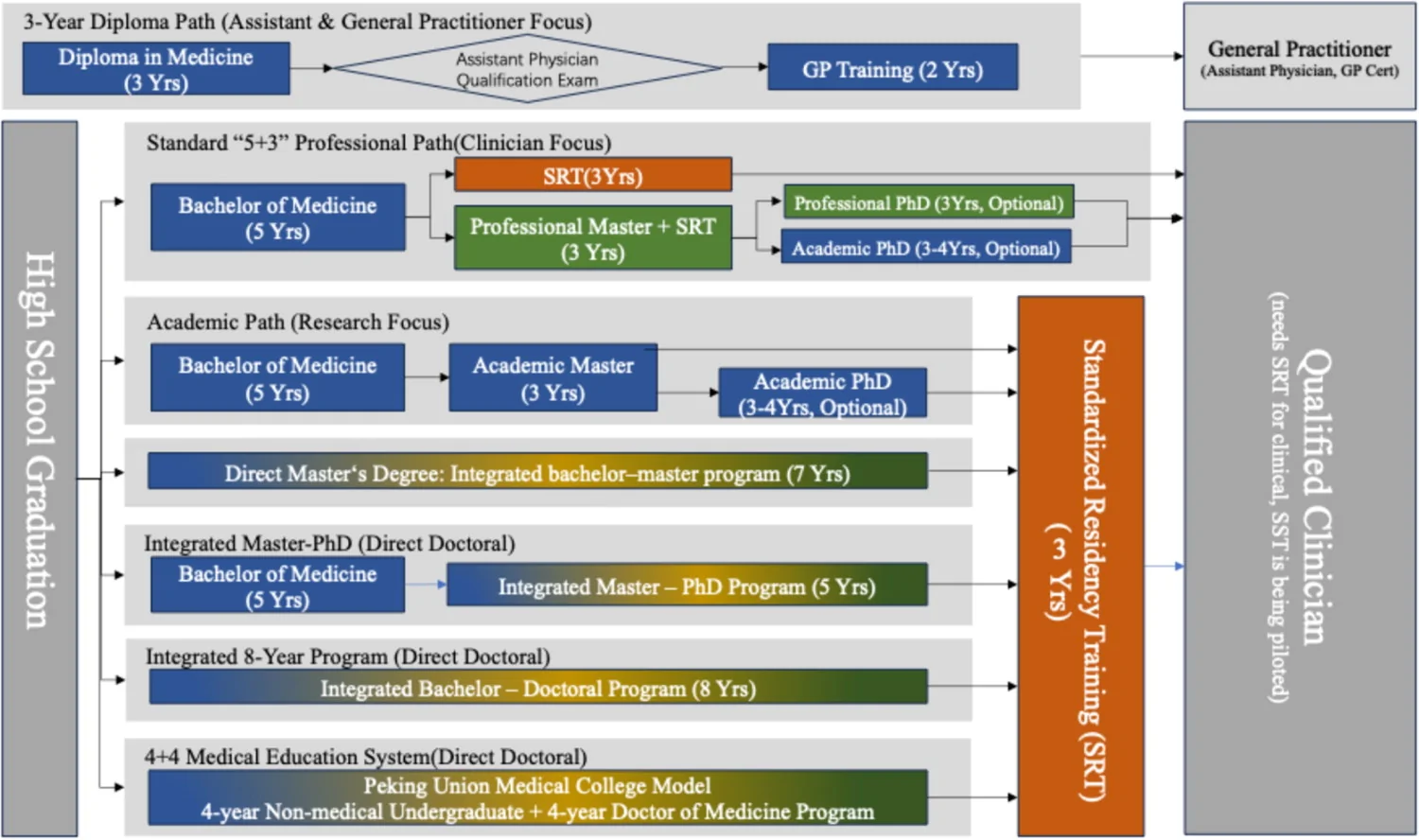
Pathways in Undergraduate and Postgraduate Medical Education in China.

Since the end of the twentieth century, China’s medical education system has undergone significant reforms, establishing a relatively comprehensive pathway from undergraduate to postgraduate education, residency training, and specialty training [15]. The current system includes a 3-year junior college medical program, a 5-year medical bachelor’s degree program, a ‘5 + 3’ medical master’s degree program, a 7-year medical master’s degree program, and an 8-year medical doctoral degree program [16,17]. In 2018, Peking Union Medical College launched the ‘4 + 4’ program, allowing outstanding university graduates from top global institutions with diverse academic backgrounds to pursue a career in medicine [18]. This section provides an overview of the structure and key characteristics of the Chinese medical education system, focusing on the core elements of undergraduate education, the postgraduate system, SRT, and SST (Figure 2). It highlights the positioning and role of each stage in the training of medical professionals, laying the foundation for a macro-level analysis of postgraduate nephrology education in subsequent sections.

### 2.1. Undergraduate medical education

Undergraduate medical education in China has developed a framework primarily based on multiple educational systems, serving as the foundational stage for training medical professionals. It primarily admits high school graduates. The curriculum is centered on foundational biomedical sciences, clinical medicine, public health and preventive medicine, and the humanities and social sciences. It is structured through a combination of required and elective courses with both horizontal and vertical integration, aligning basic and clinical knowledge while integrating theory with clinical practice. This structure systematically develops students’ core knowledge, clinical competencies, health and societal awareness, and professional attributes, ensuring effective continuity with postgraduate medical education [19]. The curriculum typically includes two years of foundational biomedical science followed by three years of hospital‑based clinical education and training, during which students study core clinical disciplines and complete comprehensive internship rotations to consolidate clinical competence. Upon conferral of the medical bachelor’s degree, graduates enter the standardized resident training program as resident trainees. After one year of training, trainees are eligible to sit for the National Medical Licensing Examination. Upon passing the examination, they are granted licensure to practice as resident physicians. The three‑year SRT program is a mandatory requirement for junior doctors before they can obtain independent clinical qualifications, regardless of the specialty they choose in the future [20].

### 2.2. Postgraduate education system

The postgraduate education system plays a key role in China’s medical education. After completing undergraduate medical education, China’s medical postgraduate education progresses into a hierarchical training stage, divided into three levels: academic master’s, professional master’s, and doctoral programs. The duration of each program is 3 years, 3 years (in conjunction with SRT) and 3–4 years, respectively. The core goal is to cultivate research-oriented or clinically applied talent in the medical field, meeting the diverse demands for high-level professionals in the healthcare industry [21]. Postgraduate degrees are divided into two categories: academic and professional. The former focuses on research training, while the latter integrates clinical practice. Professional master’s students are required to participate in SRT to enhance their clinical application skills [22].

- **Academic Master’s Degree**: The core focus is on medical research capability development. The primary candidates are undergraduates, with some clinical professionals also eligible to apply and selection is determined through the national unified examination followed by an interview conducted by the respective institutions. The training process emphasizes research training in the fields of basic or clinical medicine, with courses covering research methodology, literature review, and cutting-edge developments in the field. The graduation requirement is the completion of an original academic thesis, which must be published in a core journal or meet the academic standards set by the institution, with specific requirements determined by each university’s regulations.
- **Professional Master’s Degree**: The core focus is on enhancing clinical practice skills, closely integrated with SRT. The ‘Four-in-One’ system is implemented, meaning that postgraduates receive a master’s degree certificate, a graduation certificate, a medical practitioner qualification certificate, and an SRT completion certificate. The primary candidates are undergraduates. The training process emphasizes the development of clinical skills, covering core clinical departments such as internal medicine, surgery, and emergency medicine. The graduation requirement is that the thesis must meet the academic standards set by the institution [23].
- **Doctoral Education**: This is the highest level of medical talent development, divided into academic doctoral (PhD) and professional doctoral (MD) programs. PhD students are required to complete high-level research projects and publish papers indexed in SCI. Some institutions require MD students to complete SST, with graduation assessments considering both clinical skills and research achievements [24].

These reforms have promoted structural unification and policy-level standardization of postgraduate medical training in China. Nevertheless, challenges persist in the consistency of curriculum design, assessment mechanisms, supervision of training quality, and equitable resource distribution across training sites [24–26].

The training model for medical postgraduate students in China follows the single-mentor system, where one mentor is responsible for guiding one student from enrollment to graduation. This can lead to an uncoordinated development of students’ research capabilities and clinical practice skills [27]. In response, the Ministry of Education issued a document titled Development Plan for Professional Degree Postgraduate Education, which encourages the implementation of a dual tutorial system (DTS) for postgraduate students pursuing professional degrees, integrating clinical experience with academic mentorship [28]. In the United States, it was Case Western Reserve University the first to establish an integrated MD-PhD program, which emphasized flexibility and self-directed learning [29]. Graduate medical education (GME) in China has also piloted MD–PhD integrated training models, aiming to strengthen the integration of clinical training and academic research. The combined MD-PhD program, representing the highest level of medical and scientific training, is essential for developing future physician-scientists in China [30]. At present, such MD–PhD programs remain limited in scale and are primarily available at a small number of top-tier medical universities and research-intensive institutions (e.g. ‘Double First Class’ universities). These elite positions are extremely competitive [24]. China’s medical graduate education has reinforced the concentration of high-quality resources and innovation in training models through policies such as the National Medical Talent Training Base and support for disciplines at Double First Class universities, gradually aligning with international standards for medical graduate education. A list of “Double First-Class” universities relevant to medical education is provided in Supplementary File.

### 2.3. Standardized residency training (SRT)

SRT is a core component of China’s medical education system, targeting medical graduates with a bachelor’s degree or higher, as well as physicians who have been practicing clinically but have not undergone standardized training. Through standardized clinical rotations, this training transforms the knowledge acquired during academic education into the ability to work independently as a clinician. It is a critical stage in the transition from student to qualified clinical physician, ensuring that newly graduated doctors possess standardized clinical competencies. Since 2013, China has fully implemented the SRT system, which requires medical graduates to undergo three years of systematic clinical training after completing their formal education [31]. Training bases are primarily located in tertiary hospitals, which must undergo national or provincial evaluations to ensure they have sufficient faculty, equipment, and case resources. These hospitals are responsible for overseeing rotation-based teaching and the daily management of residents. Taking internal medicine residency training as an example, residents are required to rotate through departments such as pulmonary medicine, cardiology, gastroenterology, nephrology, emergency medicine, and critical care. Each rotation lasts between 2 and 6 months, during which residents must complete a designated number of case management tasks and skill-based procedures [32]. SRT represents one of the most standardized components of China’s medical training system, with nationally defined rotation requirements, training duration, competency benchmarks, and accreditation criteria for training hospitals.

Although SRT has been implemented for over a decade, significant challenges remain in the management of CKD and acute kidney injury (AKI) in China. These challenges include insufficient knowledge and training systems, a lack of clinical guidelines, and inadequate multidisciplinary collaboration [33]. Survey evaluations indicate that learners’ perspectives highlight barriers and facilitators to professional development, such as resource constraints and insufficient integration with clinical practice [34,35]. While the standardized process has addressed historical inconsistencies, there is still a need to strengthen supervision mechanisms, particularly in the areas of assessment and resource management [22,36,37]. In recent years, residency training reforms have increasingly focused on quality enhancement. Efforts have been made to strengthen faculty by requiring supervising physicians to hold at least the title of attending physician and undergo formal training in teaching skills. Additionally, the alignment of residency training with clinical needs has been emphasized. For instance, directed residency programs have been implemented to expand the enrollment of general practitioners and pediatricians, addressing the shortage of healthcare professionals in primary care settings. These programs are supported by policies such as tuition subsidies and employment preference. Furthermore, resources such as ‘remote teaching platforms’ and ‘simulation-based training centers’ have been introduced to improve the quality of residency training in underdeveloped regions, gradually reducing regional disparities in clinical competencies [38].

### 2.4. Standardized specialty training (SST)

SST represents an advanced phase of SRT, aimed at physicians who have either obtained a residency completion certificate or hold the title of attending physician or higher. This stage focuses on developing specialized competencies in specific medical domains to foster the advancement of subspecialties. Within the contemporary GME framework, this phase is synergistically combined with the Clinical Doctorate (MD) program. The National Health Commission (NHC) has established some training standards, which outline objectives, rotation schedules, and assessment criteria for each subspecialty. Currently, SST programs are primarily conducted within tertiary hospitals, following a ‘5 + 3 + X’ hierarchical training framework. This model involves 5 years of undergraduate medical education, 3 years of SRT, followed by 2–4 years of SST, tailored to the specific requirements of each subspecialty [31]. The Chinese medical education system, from undergraduate to subspecialty training, is centered around standardization, integrating clinical practice with scientific research. However, challenges remain, including system complexity, resource imbalances, and insufficient research. Future reforms must further optimize the system based on a balance between international best practices and domestic needs.

## 3. Postgraduate nephrology training in China

Postgraduate education in nephrology in China, as a subspecialty branch of medical postgraduate education, plays a crucial role in training highly qualified professionals with both clinical reasoning and research competencies for nephrology departments, hemodialysis centers, and research institutions at various levels of the healthcare system. The following section analyzes the core components of postgraduate education in nephrology in China from four dimensions: curriculum design, clinical rotations and teaching, research training, and assessment and evaluation.

### 3.1. Curriculum design and core competency development

In most tertiary teaching hospitals, postgraduate nephrology curricula generally consist of two core components: professional theory courses and competency-oriented courses. The professional theory courses focus on core nephrology diseases and techniques, including renal physiology, blood purification technologies, and kidney transplant management, closely following both domestic and international guidelines. The competency-oriented courses, on the other hand, emphasize clinical skills and research, covering practical topics such as evidence-based medicine and research statistics. Previous studies have reported that provinces in underdeveloped regions, including Qinghai (26.7%), Gansu (47.0%), and Heilongjiang (29.1%), exhibited markedly lower dialysis adequacy rates compared with developed provinces such as Beijing (71.0%), Jiangsu (68.2%), and Zhejiang (84.0%) [39]. These disparities not only reflect inequalities in dialysis service delivery, but also suggest broader gaps in professional competency development and training across regions [40]. Nephrology education should incorporate interactive formats such as online modules and flipped classrooms to enhance communication skills and professional competencies [41,42]. This approach may offer valuable insights for optimizing curricula in China; however, its implementation would still require localization and contextual adaptation [27].

### 3.2. Clinical rotation and practical teaching features

Clinical rotations in China leverage the resources of nephrology wards, hemodialysis centers, kidney transplant units, and related auxiliary departments within tertiary hospitals to form a structured system, integrating core departmental with subspecialty-focused training. A multidimensional practical teaching approach has been developed to ensure a stepwise enhancement of clinical competencies. Research has found that the limited number of HHD patients may lead to training gaps. Simulation training accelerates the acquisition of clinical competencies by providing a structured and safe environment for skill practice and decision-making [43]. Point-of-care ultrasound (POCUS) offers numerous potential applications in nephrology; however, its implementation is hindered by limitations in faculty resources and various logistical challenges [44]. These trends may influence the design of the Chinese system to support personalized rotation pathways [27].

The features of practical teaching are reflected in three aspects:

1. **Mentor Responsibility System + Multidisciplinary Collaborative Teaching**: Each postgraduate student is assigned one clinical mentor and 2–3 subspecialty instructors, ensuring targeted and specialized teaching.
2. **Combination of Simulation Training and Clinical Practice**: This is implemented through simulation centers, where procedures such as renal biopsy simulation and emergency dialysis management exercises (e.g., simulated treatment for dialysis-induced hypotension) are conducted. This is followed by hands-on clinical practice, which helps reduce risks [45].
3. **Case-Based Learning (CBL) and Problem-Based Learning (PBL)**: These approaches focus on complex cases to guide students in analyzing diagnostic and treatment challenges, fostering the ability to independently solve problems. Some institutions also utilize telemedicine platforms, allowing students to participate in nephrology case discussions at primary care hospitals, thereby enhancing their adaptability to diverse diagnostic and treatment scenarios [46,47].

### 3.3. Research competency development

Research competency development is a crucial component of postgraduate nephrology education in China, typically integrated with the mentorship system throughout the entire training period. Based on the differences in the positioning of academic and professional postgraduate students, a categorized and targeted approach is adopted to ensure that research capabilities align with professional development needs. Postgraduate students participate in mentor-led national or provincial research projects, undertaking activities ranging from literature review and experimental design to data collection, as well as attending departmental research meetings and academic seminars. Outstanding students may also undertake sub-projects under the guidance of their mentors. Students are typically expected to acquire familiarity with basic laboratory techniques such as Western blot, PCR, immunohistochemistry, and research skills related to kidney disease models. They may also engage in clinical research projects, addressing practical diagnostic and treatment challenges. Translational research skills are also cultivated through participation in interdisciplinary studies that leverage clinical datasets, predictive modeling, and translational analytics. For example, Chinese nephrology research increasingly integrates real-world clinical data with machine learning–based predictive modeling to advance kidney failure risk stratification, illustrating the cultivation of translational research skills [48].The Kidney Tutored Research and Education for Kidney Scholars (TREKS) program, launched by the ASN, has proven highly effective in fostering students’ research interest and career development in nephrology [49]. Research in Taiwan, China, has shown that CBL across professional groups helps enhance self-efficacy and professional performance in the field of nephrology, promoting teamwork and collaborative development [50]. With the rapid advancements in medicine, the boundaries between disciplines are increasingly becoming more fluid. The integration of practical training with interdisciplinary research, coupled with a focus on enhancing research output, will likely become a central tenet of medical postgraduate education in China [27].

### 3.4. Assessment and evaluation

The assessment framework for nephrology postgraduate students spans the entire training process, adhering to the core principles of comprehensive, multidimensional, and quantifiable evaluation. It establishes a full-cycle evaluation mechanism that includes admission education, process management, and graduation assessment. The assessment not only focuses on the mastery of theoretical knowledge and clinical skills but also emphasizes the comprehensive enhancement of research capabilities, innovation awareness, and professional competence. By developing a quantitative indicator system and an information management platform, students’ learning and research trajectories are recorded in real-time, providing data support for personalized training and quality monitoring.

In terms of clinical competence, through clinical rotation assessments, case presentations, skills evaluations, and comprehensive clinical competence assessments, students’ abilities in case analysis, diagnostic decision-making, and teamwork are evaluated, ensuring they are equipped to independently address clinical issues [51]. The assessment of research competencies emphasizes the full-process development of research thinking, methodological application, and the translation of research outcomes. Postgraduate students are required to participate in the design of research topics, data analysis, and paper writing under the guidance of their mentors. Their research literacy and innovative potential are evaluated through progress reports, mid-term assessments, and academic paper publications [52]. The evaluation of professional competence is integrated throughout the entire training process, encompassing aspects such as medical ethics, communication skills, humanistic care, and lifelong learning awareness. A multi-source evaluation mechanism is formed through mentor assessments, patient feedback, and peer evaluations, promoting the development of strong professional ethics and a sense of social responsibility among postgraduate students in both clinical and research practice.

## 4. The emerging role of critical Care nephrology

### 4.1. Differences with traditional nephrology education

CCN should be described as an emerging interdisciplinary field that bridges nephrology and intensive care medicine rather than a formally recognized independent subspecialty in China. This differs significantly from traditional nephrology education models [53]. Traditional nephrology education primarily focuses on the long-term management of CKD, the pathophysiological mechanisms of glomerular diseases, and the standardized procedures for dialysis treatment. In contrast, CCN places greater emphasis on the early identification and intervention of kidney dysfunction in critically ill patients, as well as multidisciplinary collaboration in the ICU setting. This includes the management of sepsis-associated acute kidney injury (SA-AKI), multi-organ dysfunction syndrome (MODS), and the optimization of renal replacement therapy (RRT) strategies [53,54]. Globally recognized clinical practice guidelines and consensus frameworks, such as the KDIGO AKI clinical practice guideline provide standardized criteria for AKI diagnosis, staging, and management. Similarly, expert consensus and guideline interpretations for continuous renal replacement therapy (CRRT), including indications, modality selection, timing, and treatment principles, offer well-established educational content for fellows engaged in CCN training.

The teaching methods in CCN education have also undergone a significant transformation. CBL, simulation training, and multidisciplinary rounds have been widely incorporated into postgraduate training. These approaches enable trainees to develop decision-making skills and emergency response capabilities in high-fidelity clinical scenarios [34,55]. The Department of Nephrology at the First Affiliated Hospital of Nanjing Medical University found that the CBL teaching model significantly improved theoretical performance, self-directed learning abilities, and overall learning satisfaction [56]. Similarly, the School of Medicine at Zhejiang University implemented a flipped classroom (an instructional approach in which students learn core content before class and engage in active learning activities during class) combined with case-based learning (FC + CBL) approach, comparing it with traditional instructional methods. The results demonstrated that this model significantly enhanced students’ comprehension, clinical reasoning, and collaborative skills in nephrology education [57]. Artificial intelligence (AI) has been increasingly explored as a potential supportive tool in CCN education, particularly for clinical decision support, pattern recognition in AKI, and interactive learning. Recent work on AI applications in critical care has highlighted the potential to improve diagnostic accuracy, clinical reasoning, and workflow support in CCN, suggesting that AI-integrated educational approaches may complement traditional teaching and help attract more fellows to this interdisciplinary field [58].

### 4.2. CCN training in China vs. United States

Training in CCN in China and the United States differs significantly in terms of structure, duration, and assessment methods. In China, CCN education typically involves nephrologists gaining additional critical care experience in ICU settings after completing their standard residency training. While the duration of this training varies, depending largely on individual ICU rotations, there is no formal, standardized fellowship program, nor any established competency benchmarks. As a result, training outcomes can vary widely between institutions and regions. In contrast, CCN training in the United States often occurs through structured integrated training pathways that combine nephrology and critical care medicine within coordinated programs. At several academic centers, combined nephrology–critical care medicine fellowship programs are offered, generally spanning three years and incorporating formal clinical, didactic, and multidisciplinary ICU training designed to prepare fellows for certification in both nephrology and critical care medicine upon completion. These integrated tracks have been increasingly recognized as a formal pathway for developing CCN expertise in clinical practice [59]. In summary, while CCN training in China remains in a developmental stage with significant variation, the U.S. system provides a more standardized and formally recognized pathway, with clear competencies and evaluation outcomes. As CCN continues to expand in China, efforts to formalize training programs and establish structured benchmarks will be essential for improving consistency and ensuring the effectiveness of this emerging subspecialty.

### 4.3. Implications for clinical and research talent development

The introduction of CCN has profound implications for the development of clinical and research competencies in nephrology postgraduate students. The high prevalence of AKI among critically ill patients in the ICU highlights the indispensable role of specialized nephrologists [54]. In terms of clinical competence development, the educational model of CCN significantly enhances trainees’ comprehensive diagnostic and treatment abilities, as well as their interdisciplinary collaboration skills. It trains them to manage complex multi-organ dysfunction in real clinical settings. In terms of research competence, CCN provides postgraduate students with a broad platform for scientific innovation. A systematic bibliometric analysis of high-impact articles in CCN shows that, among the top 100 most cited papers identified from Web of Science, original studies predominate, with 44% randomized clinical trials and 35% observational studies, while systematic reviews and other article types are less common. These high-impact articles are concentrated in core thematic areas, including RRT/CRRT, AKI, fluid resuscitation, pediatrics, and perioperative care [53]. The mechanisms of kidney injury in critically ill states, the discovery of early biomarkers, the optimization of RRT strategies, and the interactions between the kidneys and other organs are all current hotspots in international research [60,61]. The application of artificial intelligence (e.g. generative AI) in literature reviews and patient education is seen as a novel tool that enhances research efficiency and clinical decision-making training [62,63]. By participating in translational research projects in the ICU, postgraduate students, under the guidance of their mentors, are able to complete the full spectrum of research training, from clinical observation to mechanistic exploration. This process fosters the development of rigorous scientific thinking and innovative capabilities.

## 5. Comparative analysis of nephrology education between China and Western countries

A comparative examination of nephrology training systems across China, the United States, Europe, and Australia/New Zealand reveals both converging and divergent developmental trajectories. Table 1 summarizes the core features of these models across six domains: entry requirements, training duration, competency frameworks, assessment methods, degree of standardization, and workforce outcomes. Beyond structural differences, emerging educational needs – particularly in AI – are becoming relevant to U.S. nephrology fellowship curricula. The ASN has issued a statement emphasizing AI education and training as essential to the responsible use of AI in nephrology [64]. In a 2025 multicenter survey of Mayo Clinic nephrology fellows, 76% rated AI as moderately to highly relevant to nephrology and 76% reported moderate to very high interest in targeted AI training, yet none reported any formal AI education and most had rarely or never used AI in clinical or research activities [65]. In the same year, the Mayo Clinic reported the use of a multiagent, AI–human collaboration framework to iteratively design and refine a nephrology fellowship POCUS curriculum, which was subsequently validated by expert reviewers [66]. Clinician-led ‘Vibe Coding’ represents an emerging AI-assisted strategy for translating static nephrology education content into interactive digital learning tools [67]. Therefore, alongside the Accreditation Council for Graduate Medical Education (ACGME) milestone-based competency training, structured AI literacy may play an important role in nephrology fellowship training. AI adoption in nephrology education in China is currently low and mostly experimental.

**Table 1.** Comparison of nephrology training models in China, the U.S., Europe, and Australia/New Zealand.

Region	China	United States	Europe	Australia / New Zealand
Entry requirements	Medical degree + national licensing; entry into SRT then SST (5 + 3 + X).	Completion of ACGME internal medicine residency + ABIM eligibility.	Medical degree + core training; entry into national nephrology programs.	Completion of RACP basic training + competitive selection.
Training length	∼8–12 years, depending on integration of postgraduate degree training and/or SST.	∼9 years, with additional time for subspecialty training in some tracks.	∼9–12 years, variable by country and national training structure.	∼10–11 years, including medical degree, RACP basic training, and advanced nephrology training.
Competency frameworks	National frameworks emerging: 5 + 3 + X	ACGME Milestones	UEMS European Training	RACP curricula
Assessment methods	Rotation evaluations, procedure logs, workplace-based assessments, national exams.	Workplace-based assessment + ABIM certifying exam.	Workplace-based assessments, logbooks, national exams.	Workplace-based assessments + RACP exams.
Standardization level	Increasing national standardization but notable regional variation.	High national uniformity under ACGME/ABIM standards.	Moderate harmonization; implementation varies widely.	High consistency under RACP governance.
Workforce outcomes	Rapid workforce growth but persistent maldistribution and shortages [68].	Geographic disparities and recruitment challenges, with projected shortages in many specialties and regions [69].	Widespread shortages and reliance on foreign‑trained professionals [70, 71].	High-quality training systems, but workforce shortages in rural areas and reliance on skilled migration [72, 73].

Training length: Approximate duration from entry into medical school to independent nephrology practice; RACP: Royal Australasian College of Physicians.

The pathways to becoming a nephrologist differ fundamentally between China and Western countries, reflecting distinct educational philosophies and workforce strategies. In China, medical postgraduates typically complete a five-year undergraduate medical program, followed by SRT, after which they may enter nephrology practice directly. Although SST in nephrology is being progressively established, formal subspecialty certification is not yet universally mandatory nationwide. In contrast, the United States adopts a prolonged and sequential training pathway. Trainees must first complete undergraduate education, medical school, and a three-year internal medicine residency, followed by a dedicated nephrology fellowship accredited by the ACGME. Further subspecialization – such as transplant nephrology, onconephrology, or CCN – is pursued through additional fellowships or integrated training tracks in selected centers. Recent evidence from the United States indicates a decline in nephrology fellowship interest, with nephrology filling only about 66% of available fellowship positions in the 2024 match, a considerably lower rate compared with many other internal medicine subspecialties [74].

A distinctive feature of China’s nephrology training system is the parallel development of professional and academic postgraduate degree tracks, which differ substantially in curricular structure and training objectives. Academic master’s and doctoral programs primarily emphasize research methodology, hypothesis-driven investigation, and scholarly output, with graduation requirements typically centered on original research publications. In contrast, professional master’s and doctoral programs are explicitly designed to integrate structured clinical training with degree education. Trainees in these programs are required to complete SRT (and, in some institutions, specialty training), while simultaneously fulfilling coursework in clinical nephrology, evidence-based medicine, medical ethics, and research literacy. Clinical responsibilities constitute a substantial component of training, with rotations in nephrology wards, dialysis units, and intensive care settings forming the core experiential framework.

### 5.1. Nephrology education models in Europe and the United States

In the United States, nephrology postgraduate education is managed by the ACGME, and its training system emphasizes standardization and competency-based assessment to ensure the quality and traceability of specialty training. The framework of ACGME Milestones 2.0 emphasizes the integration of knowledge, skills, and professional attitudes in training. It advocates for the use of diverse assessment tools and structured learning scenarios to enhance the transparency and educational value of evaluation, although evidence regarding its impact on overall evaluation workload remains mixed [75]. Certifying examination pass rates appear to be associated with various program-level factors, but these relationships are multifactorial and non-causal [62]. Recent literature has suggested incorporating Comprehensive Kidney Management (CKM) into the core competency module and highlighted the lack of standardized curricula and assessment criteria in current training. This has led to inconsistencies in competency evaluation [77]. The ACGME model has facilitated the continuous improvement of education. Some U.S. programs have developed integrated training pathways that combine nephrology with oncology or critical care, allowing fellows to acquire dual competencies within a structured timeframe.

Nephrology education in Europe is regulated by the Union Européenne des Médecins Spécialistes (UEMS), which establishes unified training standards. Through its accreditation body, the European Accreditation Council for Continuing Medical Education (EACCME), UEMS promotes high-quality training and continuing professional development (CPD/CME), ensuring that trainees remain at the forefront of both clinical practice and research. The UEMS has developed detailed European Training Requirements (ETR) to facilitate the coordination and standardization of training systems across member countries [78]. However, economic and resource disparities between European countries, particularly in Central and Eastern Europe, may affect research output and the scope of training coverage [79,80]. Despite a shared European framework, implementation and specific training components may differ across countries.

Australia and New Zealand adopt a joint training model, managed by the Australia and New Zealand Society of Nephrology (ANZSN), which implements a standardized curriculum and assessment system to facilitate cross-regional training. This model emphasizes mentorship and practice-oriented learning methods. The training system evaluates the correlation between the number of trainees and clinical experience, with research indicating that trainees are generally satisfied with most training components, but there are gaps in specific clinical experiences, such as the management of complex cases. Additionally, early career paths show a trend toward diversification [81]. By assessing trainees’ experiences and trends, this model helps optimize resource allocation. However, limitations remain, including a relatively small workforce size, variable exposure to highly complex cases across training centers, and uncertainties in post-training employment, underscoring the influence of institutional scale and regional resource availability on training experience.

### 5.2. Training shortages in Africa

Despite substantial progress in nephrology education in high-income countries, the most pronounced training gaps remain in low-income regions, particularly in sub-Saharan Africa. Approximately 85% of the global AKI burden occurs in low- and middle-income countries, and overall AKI mortality in sub-Saharan Africa is high, with reported rates of ∼32% in adults and ∼34% in children. When access to indicated RRT is lacking, mortality has been observed to rise to ∼86% in adults and ∼73% in children, underscoring that limited access to RRT and critical care resources is strongly associated with worse AKI outcomes in this region [82]. Data from the ISN indicate that many African countries have fewer than one nephrologist per million population, with limited or no accredited nephrology fellowship programs and very restricted access to kidney replacement therapies [4]. The small and unevenly distributed nephrology workforce leads to delayed diagnosis of CKD and AKI, late referral, and high mortality, especially outside major urban centers [83].

Several African countries have begun to expand nephrology training through regional centers of excellence, short-term fellowships abroad, and ISN-sponsored partnerships [84]. However, most programs still face challenges, including inadequate funding, dependence on external faculty, limited imaging and biopsy capacity, and ‘brain drain’ of trained nephrologists to high-income settings. These experiences highlight that simply establishing training positions is insufficient; sustainable nephrology education in resource-constrained settings requires integrated investments in infrastructure, multidisciplinary teams, and policy recognition of CKD as a health priority. For China – where nephrology services remain unevenly distributed between urban tertiary hospitals and rural or less developed regions – African experience underscores the importance of targeted capacity-building strategies in underserved areas, accompanied by regional training hubs and tele-education platforms.

### 5.3. CKD workforce issues in India

India faces a gradually rising burden of CKD. A systematic review and meta‑analysis of community‑based studies in India reported an increase in CKD prevalence from 11.12% during 2011–2017 to 16.38% between 2018–2023 among individuals aged ≥15 years, along with significant rural–urban differences [85]. This growing disease burden occurs in the context of a nephrology workforce that remains insufficient relative to clinical need, with an estimated ∼2,600 practicing nephrologists nationwide, corresponding to approximately 1.9 nephrologists per million population – a critically low ratio when contrasted with high-income settings. This imbalance contributes to delays in diagnosis, suboptimal CKD management, and limited capacity for comprehensive care, particularly in rural and underserved regions [86].

India’s capacity to provide effective CKD care at the primary care level is influenced by multiple system-level barriers in access and workforce distribution. Qualitative research conducted in rural Indian communities identified a range of factors that may limit early CKD care, including relatively low awareness of CKD among patients and some primary healthcare providers, shortages of skilled healthcare personnel, fragmented referral pathways to specialist services, and limited availability of essential diagnostics and medicines. These conditions are linked with difficulties in achieving timely identification and management of CKD. Approaches that have been proposed to improve access to early CKD care in underserved Indian settings include strengthening clinician training, expanding CKD-specific education for healthcare workers and communities, and enhancing care coordination through structured involvement of trained community health workers [87].

### 5.4. Differences in transplant fellowship recognition

Recognition and formal accreditation of transplant nephrology fellowship training vary across international contexts. In North America, transplant nephrology fellowships are increasingly moving toward structured accreditation: the Transplant Nephrology Fellowship Training Accreditation Program (TNFTAP) provides program-level accreditation and standardized documentation that supports eligibility to lead kidney transplant programs, and efforts by professional societies are underway to secure formal the ACGME recognition of transplant nephrology as a distinct accredited subspecialty. Recent ASN/AST task force activities report progress toward finalized ACGME program requirements, which would standardize training expectations and potentially enhance formal subspecialty recognition, funding eligibility, and visa support for trainees in the United States and Canada [88]. In contrast, many regions – including China – lack globally harmonized accreditation systems specific to transplant nephrology. Fellowship recognition in these settings typically relies on national or institutional certification frameworks rather than universally accepted subspecialty accreditation, which can limit comparability of training experiences and hinder international mobility of transplant nephrology specialists [7]. These discrepancies reflect differences in clinical workforce structures and subspecialty recognition, highlighting the need for international collaboration to harmonize standards in transplant nephrology education and certification.

### 5.5. International CKD/AKI initiatives

Several international initiatives play a pivotal role in advancing the understanding, prevention, and management of CKD and AKI globally by promoting evidence‑based practice and cross‑border collaboration. The ISN leads key global efforts, such as the ISN Global Kidney Health Atlas and collaborative toolkits that synthesize the best available evidence to support clinicians and health systems in implementing early CKD screening and management initiatives worldwide [89]. KDIGO is a globally recognized nonprofit organization that develops and implements evidence‑based clinical practice guidelines for CKD, AKI, and other kidney conditions. These guidelines translate global scientific evidence into practical clinical recommendations adopted by healthcare professionals worldwide to improve patient care [90]. Consensus statements from nephrology societies, including the ISN, the ASN, and the European Renal Association (ERA), call for the inclusion of kidney disease in the global public health agenda, highlighting the need for coordinated international action to reduce morbidity and mortality [91]. These initiatives highlight the importance of harmonized guidelines, capacity‑building efforts, and global policy engagement to improve kidney health outcomes and support workforce development, especially in low‑ and middle‑income regions where the burden of CKD and AKI is increasing.

### 5.6. Innovative practices and limitations in China’s nephrology education

Under the framework of reform, China’s nephrology postgraduate education has demonstrated several innovative practices, but it still faces certain limitations. Currently, the Chinese medical education system is undergoing significant reforms, including the establishment of national training standards and the selective implementation of MD-PhD integrated programs, aiming to achieve an organic integration of clinical training and research competency development. Compared with Western competency-based training systems, China’s nephrology education remains less standardized at the level of curriculum structure, competency-based assessment, and outcome evaluation, despite the presence of nationally mandated training frameworks [92]. A study has shown that Chinese physicians have deficiencies in knowledge and practical skills in managing diabetic kidney disease (DKD), with one contributing factor being the lack of formal follow-up nephrology training [93]. In addition, uneven resource distribution and an underdeveloped continuing education system have led to training burdens and assessment challenges, making it difficult to fully integrate international standards [94].

China’s nephrology education can draw on the experiences of Western models. For example, the ACGME milestone assessment provides a standardized framework, the UEMS continuing education system promotes professional development, and the experience evaluation models from Australia and New Zealand help optimize clinical resource allocation [76,78,81]. However, China’s training system faces limitations, such as cultural differences and the lack of a unified assessment system, which have impacted the full adoption of Western models [24,94]. Future reforms should be tailored to local needs in order to drive further advancements in nephrology education.

## 6. Regional landscape and centers of excellence

The overall prevalence of CKD in China is 10.8%. Compared to other regions, the northern and southwestern areas have a higher prevalence of CKD [95]. A nationwide survey in China reported that only 25.3% of hospitalized patients underwent ≥2 serum creatinine measurements, resulting in an AKI missed-diagnosis rate of 74.2%, delayed diagnosis in 17.6% of recognized cases, and nephrology consultation in only 21.4% of patients. Among those with indications for dialysis, merely 59.3% received kidney replacement therapy. Importantly, this study demonstrated significantly higher in-hospital AKI mortality in southwest China, a region characterized by limited nephrology workforce density, restricted access to diagnostic monitoring, and delayed availability of renal replacement therapies. These findings suggest that regional disparities in AKI outcomes are closely linked to constraints in healthcare resources and specialist availability rather than disease severity alone [96]. Data from the Chinese National Renal Data System (CNRDS) show a marked expansion in dialysis access in China over the past decade. Between 2012 and 2022, the number of patients receiving hemodialysis increased from 248,016 to 844,265, while peritoneal dialysis patients increased from 36,605 to 140,544. Hemodialysis remains the predominant modality, whereas peritoneal dialysis has also expanded, particularly in tertiary hospitals in economically developed regions. Although central and western regions still face relative shortages in nephrology workforce and educational resources, recent policy-driven expansion of dialysis services has contributed to improved access nationwide and partially reduced regional disparities [39]. In terms of nephrology postgraduate education, there are also significant regional disparities in China. According to recent studies, the number of nephrology specialists in East China accounts for 33.0% of the total nationwide, while other regions, particularly Central and Western China, are comparatively underrepresented [97]. In addition to the nephrology specialist distribution, broader analyses of physician density across more than 600 Chinese cities show that overall physician density increased between 2003 and 2013, but urban districts consistently had higher physician density than county-level cities [98]. This disparity is not only reflected in the number of physicians but also in differences in educational resources, research output, and the quality of clinical practice.

In North China, nephrology education is primarily concentrated in Beijing. Peking Union Medical College Hospital and Peking University Health Science Center have trained a large number of postgraduate students and specialists. The Renal Division of Peking University First Hospital (PKUFH) and China’s National Biomedical Imaging Center (NBIC) (https://nbic.pku.edu.cn) organized major nephrology research initiatives such as the Kidney Imageomics Project [99]. These institutions not only excel in teaching quality but also play a crucial role in nephrology research and clinical practice, becoming key regional and national centers for education and research. In East China, nephrology education is particularly prominent in cities such as Shanghai and Zhejiang. Ruijin Hospital in Shanghai has longstanding experience in nephrology education, particularly in specialized fields like kidney transplantation and dialysis treatment, where it has accumulated substantial teaching experience and clinical outcomes. Clinicians from this center participated in a phase 3 randomized trial comparing roxadustat with epoetin alfa in patients undergoing long-term dialysis [100]. At the Kidney Disease Center of The First Affiliated Hospital, Zhejiang University School of Medicine, researchers have examined amino acid metabolism in DKD through metabolomics studies, exemplifying the center’s interdisciplinary strengths in integrating clinical nephrology with molecular and computational approaches [101]. In Southwest China, nephrology fellows demonstrated good basic knowledge but only moderate attitudes and practice regarding vascular access training for chronic hemodialysis patients, and specific areas requiring additional education were identified to improve clinical practice and guideline-aligned management of vascular access in maintenance hemodialysis patients [102].

With the regional imbalance in healthcare resources, cross-regional collaboration has become an important means of improving the quality of nephrology education. To optimize the allocation of educational resources, several provinces have established resource-sharing platforms, effectively alleviating the issue of resource inequality caused by geographical differences. With the development of information technology, remote education and online training have become crucial methods for enhancing the quality of nephrology education in central and western China. For example, West China Hospital of Sichuan University, as the largest center for the diagnosis and treatment of complex and critical kidney diseases in Western China, not only provides extensive technical support and patient management but has also developed specialized projects such as nocturnal home automatic peritoneal dialysis (APD). As the largest peritoneal dialysis center in Western China, West China Hospital has trained a large number of nephrology professionals from across the country through specialized conferences on hemodialysis, peritoneal dialysis, and renal pathology [103]. Their teams have published studies on acute kidney injury (AKI) risk factors and biomarkers (e.g. prognostic role of neutrophil‑to‑lymphocyte ratio in septic AKI), demonstrating active clinical nephrology research in high‑impact settings [104]. These regional collaboration models have not only facilitated the sharing of educational resources but also improved the overall level of healthcare.

## 7. Career trajectories and educational development trends

### 7.1. Academic and clinical career trajectories

The career development of nephrology postgraduate students in China primarily follows two pathways: clinical physician advancement and academic research trajectories. The clinical path includes SRT, specialty physician promotion, and evaluations for titles such as Associate Chief Physician or Chief Physician, focusing on the accumulation of clinical skills and case management experience. The academic trajectory, on the other hand, involves pursuing continuous master’s and doctoral programs, MD-PhD projects, or promotion through research positions, emphasizing the development of research capabilities, project applications, paper publications, and international academic exchanges. In practice, many nephrology professionals balance both clinical and research roles, forming a dual-track clinical and research development model. However, this model faces challenges, including imbalanced time allocation between research and clinical duties, as well as inconsistencies in career advancement evaluation systems.

### 7.2. Current international collaboration

China’s nephrology postgraduate education is expanding its global perspective through various forms of international collaboration. Firstly, student exchange programs and joint training initiatives promote cross-cultural communication. Some top medical schools and hospitals collaborate with universities in Europe, the United States, or other Asian countries to conduct short-term or long-term student exchange and joint training programs, thereby enhancing clinical skills and broadening research perspectives [105]. Secondly, clinical physicians and postgraduate students participate in visiting scholar programs to study the latest diagnostic and therapeutic methods, as well as research techniques abroad. This helps bring in advanced experiences and technologies [106]. Moreover, Chinese nephrology scholars actively participate in international academic conferences, such as those organized by the ISN, thereby enhancing China’s influence in the global nephrology community [107].

## 8. Future perspectives

With the acceleration of globalization, nephrology education is facing unprecedented opportunities and challenges. The future development of nephrology education will focus on emerging technological fields and the evolving needs of the profession, particularly the growing demand for experts in digital healthcare and AI [108]. AI algorithms have the potential to analyze patient data – such as medical records and laboratory results – and support personalized educational content in nephrology training [109]. Additionally, the demand for specialized professionals in precision medicine and gene therapy is increasing. As precision medicine continues to evolve, research and treatment methods in nephrology are also advancing [110,111]. To address these changes, nephrology professionals will need to possess interdisciplinary knowledge and skills, including expertise in molecular biology, genomics, and data analysis.

China’s nephrology education should further strengthen international collaboration and academic exchange, promoting global knowledge sharing through multidimensional academic cooperation platforms. In Europe, the Nephrology Partnership for Advancing Technology in Healthcare (N-PATH) program aims to address the shortage of nephrology specialists by improving educational quality and fostering interdisciplinary collaboration [112,113]. Drawing on international experience while considering local realities, the development of targeted training and evaluation systems will help enhance the attractiveness and status of the nephrology specialty, fostering professionals capable of adapting to the rapidly changing healthcare environment.

Meanwhile, the standardization of the education system requires urgent improvement, including the development of unified training norms and assessment standards to ensure that trainees receive comprehensive theoretical knowledge and practical training [31]. Nephrology education must strengthen interdisciplinary collaboration with other medical fields, particularly internal medicine and critical care, to develop professionals with comprehensive clinical problem-solving abilities [114]. Through these measures, the quality and level of nephrology education are expected to improve progressively, potentially contributing to broader efforts in the prevention and treatment of kidney diseases.

## Supplementary Material

Supplemental Material

## Data Availability

Data sharing is not applicable to this article as no new data were created or analyzed in this study.
