# Supplementary material for: Post-graduate nephrology education in China: structure, workforce gaps, regional disparities, and the emerging role of critical care nephrology
Source: Ren Fail. 2026 Sep 22;48(1):2731711. doi: 10.1080/0886022X.2026.2731711 (PMC13600322; doi:10.1080/0886022X.2026.2731711)
Supplement: Supplementary file 1 [file IRNF_A_2731711_SM2400.docx]

**Supplementary File. “Double First-Class” Universities Relevant to Medical Education in China**

This supplementary table includes universities in the second-round “Double First-Class” initiative whose officially listed construction disciplines include medicine-related fields. For the purpose of this review on postgraduate medical education, medicine-related fields were defined as Basic Medicine, Clinical Medicine, Stomatology, Public Health and Preventive Medicine, Traditional Chinese Medicine, Integrated Chinese and Western Medicine, Pharmacy, Chinese Materia Medica, and Nursing.

| **University** | **Medicine-related “Double First-Class” construction discipline(s)** |
| --- | --- |
| Peking University | Medicine-related disciplines are autonomously determined and published by the university* |
| Peking Union Medical College | Clinical Medicine; Public Health and Preventive Medicine; Pharmacy |
| Beijing University of Chinese Medicine | Traditional Chinese Medicine; Chinese Materia Medica |
| Tianjin Medical University | Clinical Medicine |
| Tianjin University of Traditional Chinese Medicine | Traditional Chinese Medicine |
| Fudan University | Basic Medicine; Clinical Medicine; Public Health and Preventive Medicine; Integrated Chinese and Western Medicine; Pharmacy |
| Shanghai Jiao Tong University | Basic Medicine; Clinical Medicine; Stomatology; Pharmacy |
| Shanghai University of Traditional Chinese Medicine | Traditional Chinese Medicine; Chinese Materia Medica |
| Nanjing Medical University | Public Health and Preventive Medicine |
| Nanjing University of Chinese Medicine | Traditional Chinese Medicine |
| China Pharmaceutical University | Chinese Materia Medica |
| Zhejiang University | Basic Medicine; Clinical Medicine; Pharmacy |
| Shandong University | Clinical Medicine |
| Zhengzhou University | Clinical Medicine |
| Wuhan University | Stomatology |
| Huazhong University of Science and Technology | Basic Medicine; Clinical Medicine; Public Health and Preventive Medicine |
| Sun Yat-sen University | Basic Medicine; Clinical Medicine; Pharmacy |
| Jinan University | Pharmacy |
| Guangzhou Medical University | Clinical Medicine |
| Guangzhou University of Chinese Medicine | Traditional Chinese Medicine |
| Sichuan University | Basic Medicine; Stomatology; Nursing |
| Chengdu University of Traditional Chinese Medicine | Traditional Chinese Medicine |
| Naval Medical University | Basic Medicine |
| Air Force Medical University | Clinical Medicine |

***Note:** Peking University is included because it is a “Double First-Class” university with a major medical education and research system. In the second-round national list, Peking University and Tsinghua University were permitted to autonomously determine and publish their construction disciplines. This table is intended to identify medicine-relevant institutions and should not be interpreted as a list of universities that all offer an identical MD–PhD program.

**Official source:** Ministry of Education of the People’s Republic of China, Ministry of Finance, and National Development and Reform Commission. Notice on the publication of the second-round list of “Double First-Class” universities and construction disciplines (Jiaoyan Han [2022] No. 1), 14 February 2022. http://www.moe.gov.cn/srcsite/A22/s7065/202202/t20220211_598710.html.
